# Allergic Reaction to Red Tattoo Ink: A Case Report of an Anime-Inspired Tattoo in an Immunocompromised Patient

**DOI:** 10.7759/cureus.82095

**Published:** 2025-04-11

**Authors:** Axel R Marquez-Nuñez, Gil Perez-Vazquez, Juan E Abad Olmedo, Elizabeth C Ramos Lopez, Cinthia F Castro Carcaño, Ruth P Sotelo Loeza, Marvin J Benavides Maruri, Valeria L Diaz-Molina

**Affiliations:** 1 Dermatology, National Autonomous University of Mexico, Mexico City, MEX; 2 Dermatology, Centro Medico Nacional 20 de Noviembre, Instituto de Seguridad y Servicios Sociales de los Trabajadores del Estado, Mexico City, MEX; 3 Pathology, Centro Medico Nacional 20 de Noviembre, Instituto de Seguridad y Servicios Sociales de los Trabajadores del Estado, Mexico City, MEX; 4 Dermatology, Hospital General de Mexico "Dr. Eduardo Liceaga", Mexico City, MEX

**Keywords:** immunosuppression, multiple sclerosis, skin disorders from tattooing, tattoo, tattoo allergy, tattoo complication, tattoo ink reaction

## Abstract

Tattoos have become a form of artistic expression throughout human life. They have also been used in the medical field in various subspecialties for diagnostic and therapeutic purposes. The rise in this activity has been accompanied by increased cutaneous and systemic complications associated with various pigments and application techniques. Some cutaneous complications related to tattooing may include inflammatory, infectious, and proliferative forms. It is well-known that there are relative contraindications for patients with systemic or skin conditions, which pose a higher risk for complications following tattoo application. One relative contraindication may be immunosuppression caused by a chronic degenerative disease or medications that compromise immunity. This immunosuppression can be either temporary or permanent. We present an atypical case of a patient who experienced a complication secondary to red tattoo ink within the context of immunosuppression due to treatment for his underlying disease.

## Introduction

Tattoos have been a diverse form of body expression, and over the past few decades, they have gained popularity across all social strata and at different stages of life. This increased popularity has led to greater interest in researching the health risks associated with tattoo application [1]. Patients with chronic diseases represent a demographic that is particularly interested in getting tattoos, raising important medical questions about whether they are exposed to the same complications as the general population [2]. Whether due to the underlying disease or the medications used to control it, the significance of reviewing this topic is undeniable. Although it may be perceived as trivial, it should not be overlooked, as 18% of the adult population in Western countries has one or more tattoos [3]. We present the case of a 31-year-old man with a history of multiple sclerosis who, two years after his last tattoo, experienced an increase in volume and itching in the area with red ink, which may be attributed to the application of glatiramer acetate.

## Case presentation

A 31-year-old male patient with a history of relapsing-remitting multiple sclerosis, evolving over 15 years and treated with glatiramer acetate for the past five years, presented for consultation. He reported pain, itching, and the development of an elevated plaque on the anterior surface of his right forearm at the site of the red pigment of his most recent tattoo, which he got tattooed two years ago; this lesion has been present for four months. Physical examination revealed multiple tattoos in various body areas, created with dark inks and colors (yellow, red, and blue).

**Figure 1:**
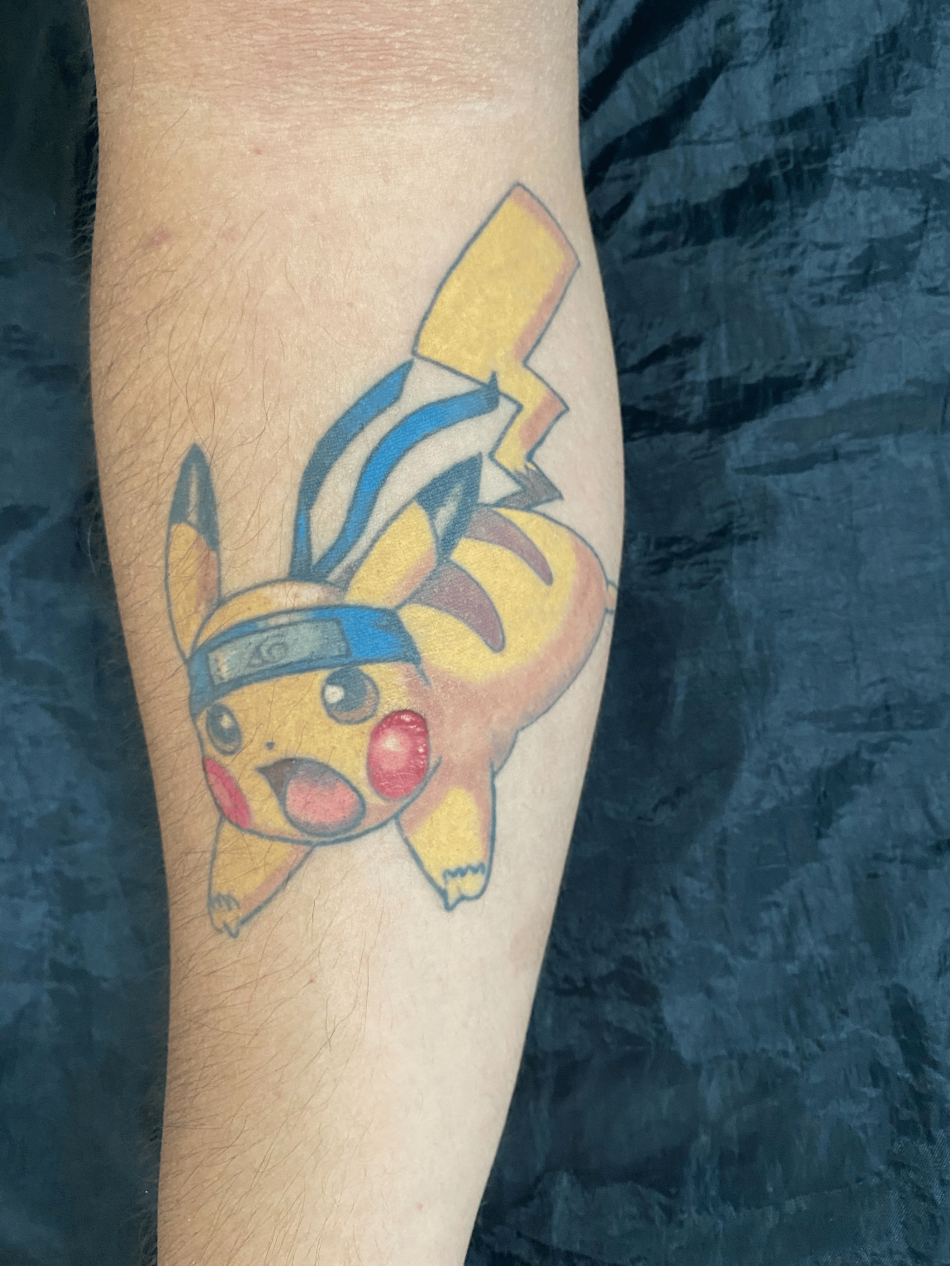
Dermatosis located at the right forearm. Two elevated plaques with a scaly surface within the red ink of the tattoo. Written informed consent to include this image in an open-access article was obtained from the patient.

Notably, in the tattoo associated with his symptoms, a dermatosis was observed, characterized by two elevated plaques with a scaly surface measuring 5 millimeters, with well-defined edges (Figure 1).

**Figure 2:**
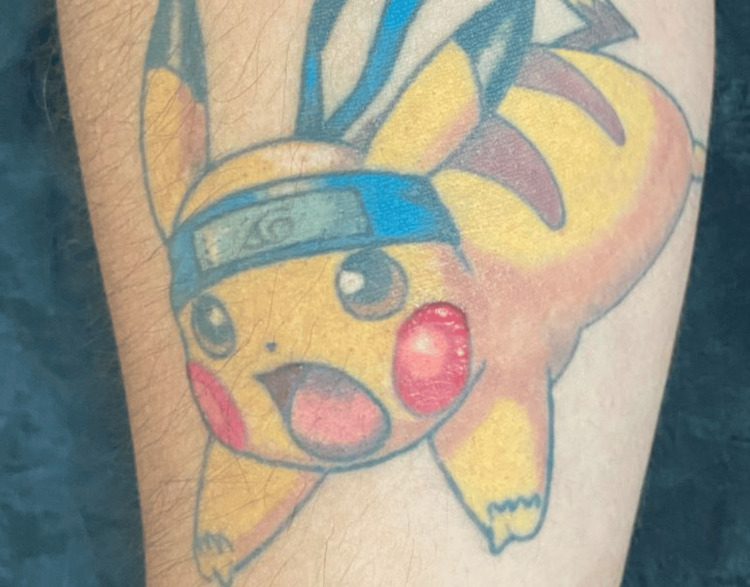
Elevated plaques with a scaly surface. Lesions are exclusively found within the red ink of the tattoo. An elevated and rough surface is observed. Written informed consent to include this image in an open-access article was obtained from the patient.

These lesions were exclusively found within the tattoo's red ink, sparing the rest of the design (Figure 2).

**Figure 3:**
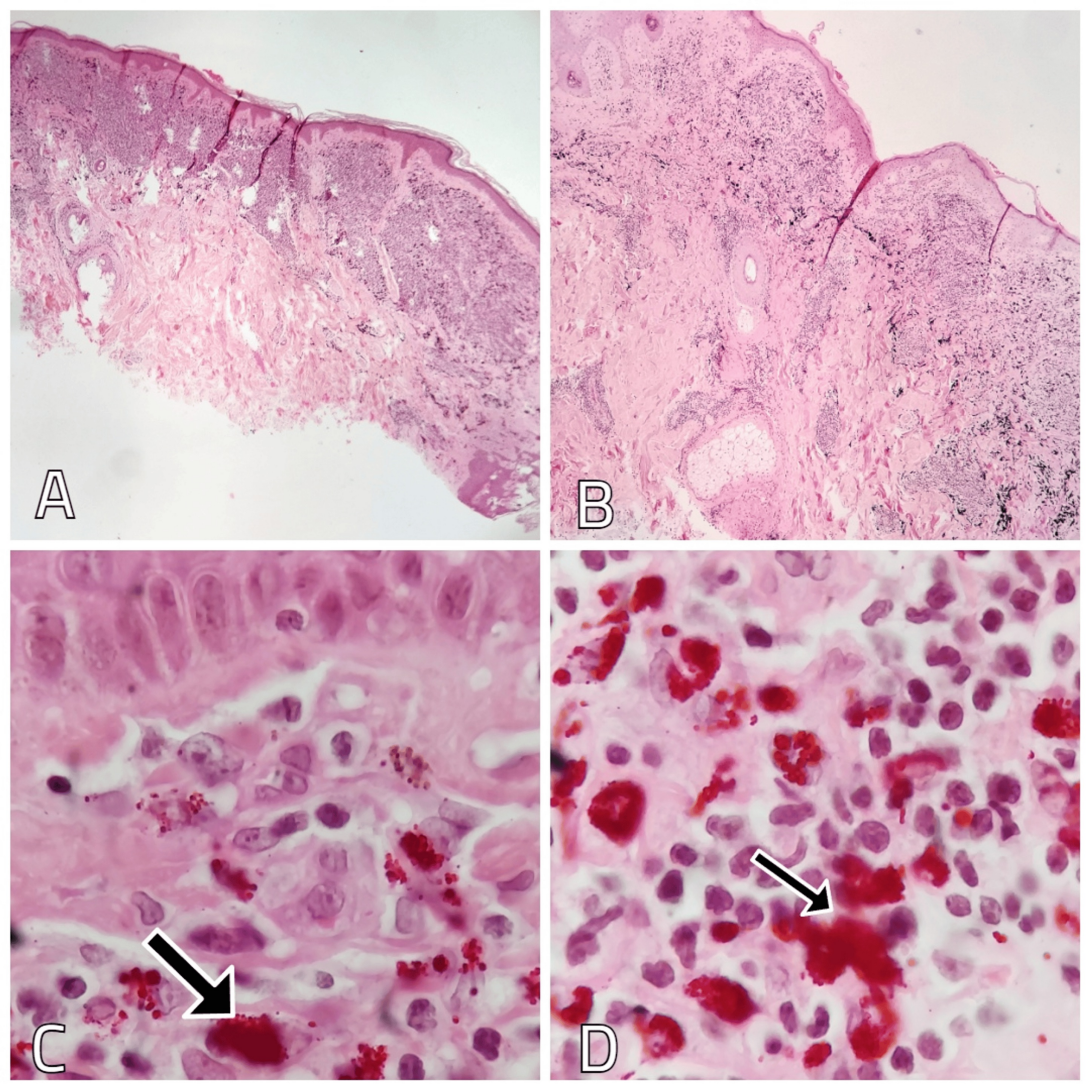
Biopsy findings of the red tattoo ink on the left forearm. A and B: Hematoxylin and eosin staining showing hyperkeratosis, areas of epidermal atrophy and inflammatory infiltrate extending through the papillary and reticular dermis. Magnification x10. C and D: Hematoxylin and eosin staining showing lymphohistiocytic-type inflammatory infiltrate and the presence of reddish pigment granules (arrows). Magnification x40.

A biopsy of the lesion showed the epidermis with compact hyperkeratosis and areas of epidermal atrophy in some parts of the specimen. The infiltrate was of a lymphohistiocytic inflammatory type, extending through both the papillary and reticular dermis, with reddish pigment granules and few lymphocytes (Figure 3).

The histopathological findings established a diagnosis of an allergic inflammatory reaction to exogenous pigment (red ink). Consequently, a high-potency topical corticosteroid treatment was initiated, leading to clinical improvement and resolution of symptoms within eight weeks.

## Discussion

Tattooing involves the introduction of exogenous pigments and/or dyes into the dermis to create a permanent design, which is now considered a form of body art [4]. Some complications that may arise after tattooing include allergic reactions to the ink, infections, and local or systemic complications. Among the complications associated with tattooing, allergic inflammatory reactions to the pigments or inks are the most common and are typically characterized by itching, chronic pain, formation of papules, nodules, and induration, generally restricted to one color within the tattoo. Red pigments are involved in the vast majority of cases, with symptoms usually appearing approximately one year after the tattoo is applied. However, the onset may range from one month to several years [1]. Given the increasing popularity of tattooing, chronic disease patients are likely to be interested in this activity and may seek advice from their treating physician to help avoid such complications.

Extensive research has been conducted in France and other Western European countries regarding tattoos, including complications associated with this practice and the restriction of hazardous substances used in tattoo and permanent makeup inks [5]. There is no statistical data on the tattooed population in Mexico, nor is there concrete legislation concerning harmful substances that may or may not be used during tattooing. Another opportunity for deeper investigation relates to local or systemic reactions that may be part of the autoimmunity triggered by tattoo pigments concerning the patient's chronic illness (whether it is a multisystem disease or one predominantly affecting the integumentary system). This is particularly relevant in cases where biologics, targeted therapies, and immune checkpoint inhibitors may be suggested as potential triggers for tattoo complications [1].

Multiple sclerosis is an inflammatory autoimmune disease of the central nervous system with an unknown etiology. Various medications are used to alleviate the symptoms caused by this condition, one of which is glatiramer acetate [6]. The mechanism of action of this drug is based on immunomodulation, wherein the response of T-cells to myelin antigens is inhibited by binding to the major histocompatibility complex [7]. The administration of glatiramer acetate may likely have a significant association with the triggering of autoimmune disorders, inducing local cytokines that can initiate inflammatory reactions in the skin [7].

When a hypersensitivity/allergic reaction to tattoo pigments and inks is clinically identified, a punch biopsy is recommended to identify different histological patterns, such as eczematous reaction, lichenoid reaction, lymphohistiocytic reaction, foreign body granulomatous reactions, sarcoidosis-like reactions, and pseudolymphoma [8]. Treatment can be complex, with high-potency topical corticosteroids recommended as the first-line therapeutic approach for 8-12 weeks [8].

Tattooing can involve a wide range of complications, yet an increasing number of individuals are drawn to the practice-whether first-timers, those looking to complement an existing tattoo, or simply seeking a new design. We know that tattoo inks are industrial products that may contain impurities and various contaminants, varying between brands, batches, and the particles tattoo artists use in their work [1]. For this reason, it is important to advise patients with known skin conditions, chronic diseases, and/or those experiencing any form of immunosuppression, either due to an underlying health issue or medication they are receiving for its management, to seek guidance from their physician regarding the possibility of getting a tattoo and the potential risks of complications in the short, medium, or long term [9].

## Conclusions

We present the case of a 31-year-old male diagnosed with multiple sclerosis, currently treated with glatiramer acetate, who is an avid client of various tattoo artists. The patient sought help for a complication secondary to the red ink of his most recent tattoo. He presented with an allergic reaction to the red pigment, which was clinically suspected and confirmed by biopsy. We emphasize the need for further studies to determine whether there is a causal relationship between immunomodulatory drugs such as glatiramer acetate and the predisposition of immunosuppressed patients to develop adverse reactions to tattoo inks.

## References

[1] Giulbudagian M, Battisini B, Bäumler W (2024). Lessons learned in a decade: Medical-toxicological view of tattooing. J Eur Acad Dermatol Venereol.

[2] Kluger N (2019). Tattooing and multiple sclerosis: A study among 445 French patients. Presse Med.

[3] Kluger N, Seité S, Taieb C (2019). The prevalence of tattooing and motivations in five major countries over the world. J Eur Acad Dermatol Venereol.

[4] Kluger N (2013). Tattooing and piercing: an underestimated issue for immunocompromised patients?. Presse Med.

[5] Kluger N (2020). Looking back at fifteen years of publications on tattoos. Presse Med.

[6] Rastkar M, Ghajarzadeh M, Sahraian MA (2023). Adverse side effects of Glatiramer acetate and Interferon beta-1a in patients with multiple sclerosis: A systematic review of case reports. Curr J Neurol.

[7] Schrempf W, Ziemssen T (2007). Glatiramer acetate: mechanisms of action in multiple sclerosis. Autoimmun Rev.

[8] Kluger N (2019). An update on cutaneous complications of permanent tattooing. Expert Rev Clin Immunol.

[9] Kluger N (2015). Contraindications for tattooing. Curr Probl Dermatol.

